# Percutaneous Mechanical Aspiration of Bioprosthetic Tricuspid Valve Vegetation and Thrombi in Drug Use-Associated Infective Endocarditis

**DOI:** 10.1016/j.cjco.2026.01.013

**Published:** 2026-02-04

**Authors:** Tiffany Furneaux, Ashar Pirzada, Frederic Paulin, Alison Greene, Scott Harris, Mohammad Almutawa

**Affiliations:** a Faculty of Medicine, Memorial University of Newfoundland, St. John’s, Newfoundland and Labrador, Canada; b Division of Cardiology, Health Sciences Centre, Newfoundland and Labrador Health Services, St. John’s, Newfoundland and Labrador, Canada; c Division of Cardiac Surgery, Health Sciences Centre, Newfoundland and Labrador Health Services, St. John’s, Newfoundland and Labrador, Canada; d Division of Radiology, Health Sciences Centre, Newfoundland and Labrador Health Services, St. John’s, Newfoundland and Labrador, Canada

**Keywords:** infective endocarditis, drug use-associated infective endocarditis, percutaneous mechanical aspiration, bioprosthetic valve, candida endocarditis, right-sided endocarditis, interventional cardiology

**A 27-year-old male patient with a history of intravenous drug use presented with recurrent fungal prosthetic tricuspid valve endocarditis and septic emboli, 6 months after a prior bioprosthetic valve replacement for methicillin-susceptible *Staphylococcus aureus* (MSSA) infective endocarditis (IE). Given his high surgical risk, he underwent percutaneous mechanical aspiration (PMA) of the valve vegetation and right atrial thrombi using a Penumbra** **(Alameda, CA)** **16F catheter system. PMA was well tolerated, and it achieved a > 50% reduction in vegetation burden, and modest improvement to the transvalvular gradient (from 11 to 6 mm Hg). This case supports expansion of PMA applications in high-risk drug use-associated IE.**

The incidence of IE is rising globally, with over 1.09 million cases diagnosed in 2019.^1^ A disturbing rise in IE-related mortality also is occurring among younger adults, driven largely by the ongoing opioid crisis.^2^ The proportion of IE cases associated with intravenous drug use (IDU-IE) nearly doubled between 2010 and 2015, and it continues to increase.^2^

*Staphylococcus aureus* remains the most commonly isolated organism in both native and prosthetic valve endocarditis in IDU-IE, whereas *Candida* species, though less common, are associated with significantly higher mortality—estimated to be around 40%.^3^ *Candida* prosthetic valve endocarditis typically develops within 2 months of valve implantation, often due to nosocomial exposure. IDU-IE affects predominantly the right side of the heart and increases the risk of pulmonary embolization.^4^

The treatment of IDU-IE poses unique challenges due to high rates of drug use recurrence and reinfection post-valve replacement. In this context, PMA may offer benefits by reducing embolic risk, lowering infectious burden, and serving as a palliative therapy in patients who are not surgical candidates.

## Clinical History

A 27-year-old man with known substance use disorder on methadone maintenance presented to the emergency department with 1 month of progressive dyspnea, cough, and weakness. He had undergone bioprosthetic tricuspid valve replacement (Abbott [Chicago, IL] Epic Plus #33) for MSSA endocarditis in October 2024, complicated by cavitary lung lesions and a pelvic abscess. He later admitted to relapse into intravenous cocaine use.

Laboratory results showed anemia (hemoglobin, 99 g/L), elevated international normalized ratio (2.84), and hyponatremia (sodium, 128 mmol/L). Blood cultures obtained on April 7, 2025 were positive for *Candida dubliniensis*. Computed tomography of the chest showed cavitating pulmonary nodules, pulmonary emboli, bilateral lower lobe consolidation, and retroperitoneal lymphadenopathy. Transesophageal echocardiography (TEE) revealed a large (2.3 x 0.7 cm) mobile vegetation on the prosthetic tricuspid valve, increased leaflet echogenicity, moderate-to-severe tricuspid regurgitation (TR), and tricuspid stenosis (mean gradient, 11 mm Hg).

## Diagnostic Methods

Computed tomography of the chest (April 24, 2025) showed the following:

• Worsening bilateral lower lobe consolidation; and
• Cavitating nodules consistent with septic emboli.
TEE (April 30, 2025) showed the following:
• Normal left ventricular function;
• Mild right ventricular dilatation and low-normal systolic function;
• Bioprosthetic tricuspid valve with large mobile vegetation (∼ 2.3 x 0.7 cm);
• Moderate-to-severe TR and stenosis (mean gradient, 11 mm Hg at 100-110 beats per minute); and
• Severe pulmonary regurgitation.

## Procedure and Treatment

After multidisciplinary discussions, the patient was deemed unsuitable for repeat surgical intervention due to active substance use disorder, high operative risk, and prosthetic involvement. PMA was recommended as a therapeutic option to reduce vegetation burden and stabilize the patient.

Fluconazole at 400 mg orally daily was initiated. On April 30, 2025, the patient underwent PMA using a Lightning Flash 2.0 System. The system offers a simpler setup without a perfusionist and activates aspiration with a simple switch engine rather than continuous suction. Built-in pressure sensors provide real-time audio and visual flow feedback. Its smaller catheter profile improves maneuverability and reduces potential complications. Limitations include modest aspiration force, the need for direct vegetation contact, and the absence of a blood-return mechanism.

## Procedure Details

• The patient was electively intubated prior to the procedure to facilitate TEE-guided aspiration.
• The right internal jugular vein was occluded; the procedure proceeded via dual femoral venous access.
• Intracardiac echocardiography and TEE confirmed vegetation and right atrial thrombus.
• A 17F Penumbra sheath (Penumbra) was inserted into the inferior vena cava, and an aspiration catheter was advanced under fluoroscopy and intracardiac echocardiography.
• Moderate vegetation was debulked.
• An additional left internal jugular approach and snaring from the inferior vena cava were attempted, but adherent vegetation limited complete removal.
• Total blood loss was 400-500 mL; one-unit packed red blood cells wastransfused.
• No periprocedural complications occurred.

## Results

**Figure 1:**
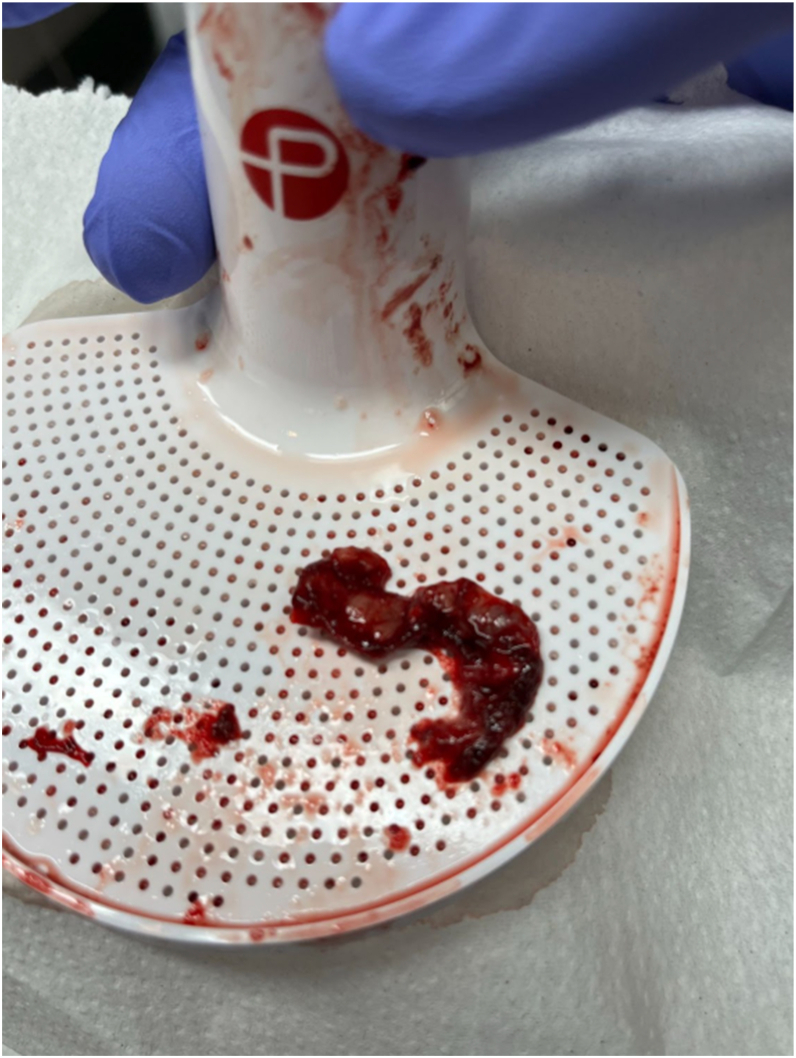
Aspirated vegetation following percutaneous mechanical aspiration using the Penumbra Lighting Flash 2.0 (Alameda, CA) Catheter System. Its appearance is consistent with friable, fungal infective endocarditis vegetation. Cultured aspirated material was found to be *Candida tropicalis*, suggesting polymicrobial fungal infection.

**Figure 2:**
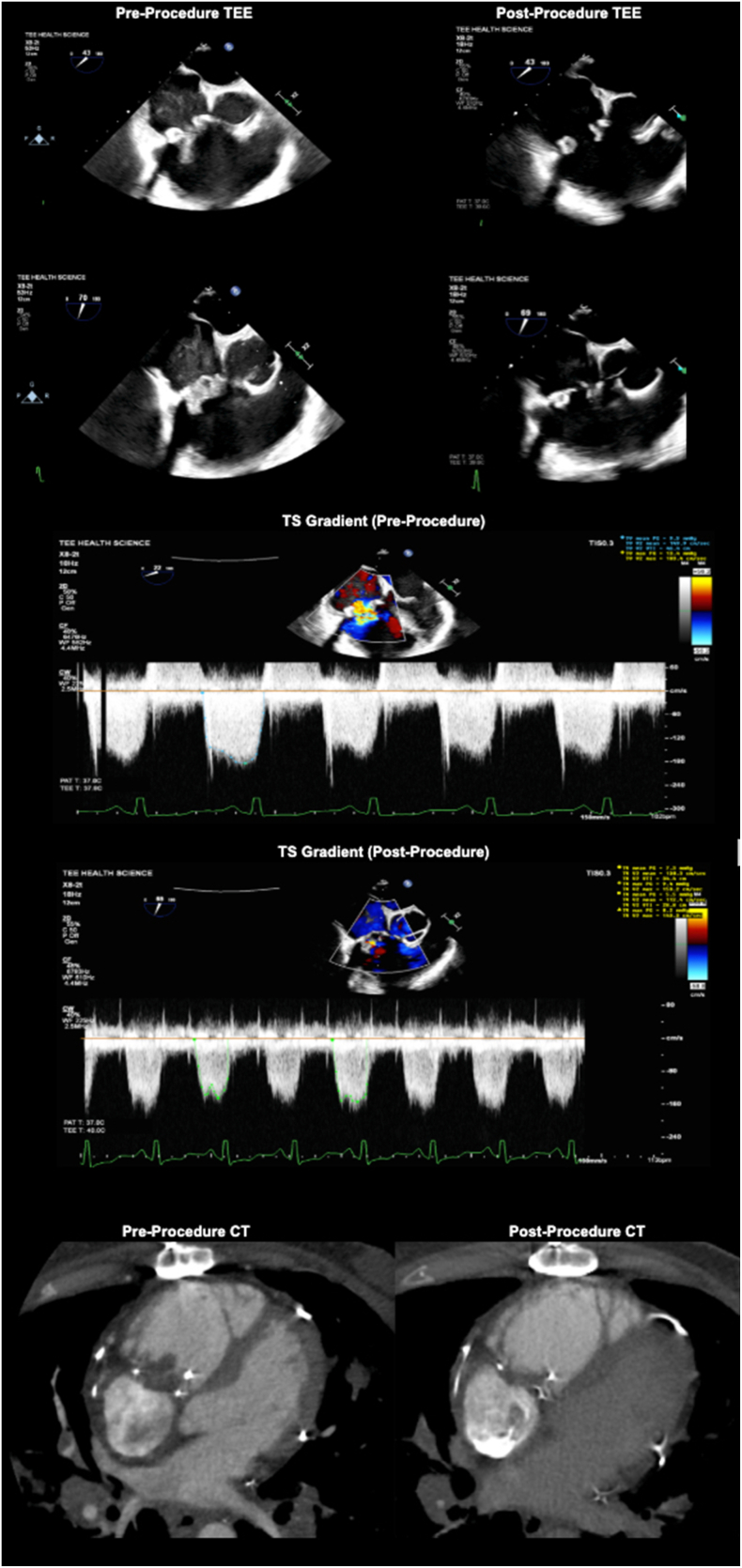
**Top panels:** Pre- and post-procedure transesophageal echocardiography (TEE) visualizations showing reduced mobility and size of the vegetation on the bioprosthetic tricuspid valve. **Middle panels:** Continuous wave Doppler. **Bottom panels:** Computed tomography (CT) demonstrating the vegetation burden (**left**) before and (**right**) after percutaneous mechanical aspiration. TS, tricuspid stenosis.

Post-procedural TEE findings were as follows (Figs. 1 and 2):

• Vegetation was reduced by ∼50% (∼1.2 x 0.5 cm), no longer pedunculated.
• Tricuspid valve mean gradient was reduced to 6 mm Hg at 100-110 beats per minute immediately post-aspiration.
• TR severity was unchanged (moderate-to-severe).

Clinical improvement included better oxygenation (oxygen saturation 92% on room air), resolution of fever, and improved exercise tolerance. Blood cultures remained negative.

The patient initially improved with antifungal therapy and mechanical aspiration, achieving negative cultures, smaller vegetations, and better gradients. The goal was to debulk the vegetation to reduce stenosis of the tricuspid valve rather than cure, aiming to lower the candidemia burden and reduce right-sided heart failure as much as possible.

Despite 6 weeks of therapy and persistently negative cultures, repeat imaging showed a larger vegetation of the tricuspid valve with worsening stenosis and regurgitation. The patient admitted to ongoing drug use. With no surgical options left and progressive valve failure, he was transitioned into palliative care in August 2025.

## Discussion

This case underscores the evolving role of PMA in the management of fungal prosthetic valve endocarditis, particularly in patients with contraindications to surgery. The use of a large-bore 16F-17F Penumbra catheter enabled effective aspiration of bulky vegetations and thrombi, with symptomatic improvement. Following mechanical aspiration, the transvalvular gradient decreased modestly (from 11 to 6 mm Hg), with unchanged TR. TR severity was assessed per 2017 American Society of Echocardiography guidelines for valve regurgitation. These findings suggest partial hemodynamic improvement rather than full restoration of valve function.

The rise of IDU-IE presents a formidable clinical challenge. Conventional surgical paradigms are complicated by high rates of relapse and reinfection. In this case, PMA reduced the vegetation size by approximately 50% immediately post-procedure. No embolic events were observed during the subsequent 6 months, underscoring the potential long-term benefit of this approach in reducing embolic risk. This case supports previous literature emphasizing the safety and utility of PMA in right-sided IE.^4^^,^^5^

## Conclusions

PMA may be a safe and effective option in managing fungal prosthetic valve endocarditis in high-risk patients with drug use–associated IE. Although it is not curative, PMA offers a reduction in vegetation burden, and symptomatic improvement in select patients who are not surgical candidates. This case is the first attempt at debulking *Candida* spp. vegetation on a bioprosthetic value. Immediate results were positive; no complications were noted immediately following the procedure or at the 6-month follow-up. We successfully debulked 50% of the vegetation and reduced the embolic risk of previously pedunculated vegetation. These outcomes are not comparable to those of surgery, which should remain the gold standard for viable candidates.

This therapy requires future studies to determine the appropriate approach and timing, and its long-term clinical impact.

**Novel Teaching Points**

• This case is the first attempt at debulking *Candida* spp. vegetation on a bioprosthetic value.
• Smaller-bore aspiration systems are effective for debulking fungal vegetations.
• *Candida* polymicrobial infection can complicate prosthetic valve IE.
• PMA can bridge to future surgical or addiction-focused care in select cases.
